# Confirming a Role for α9nAChRs and SK Potassium Channels in Type II Hair Cells of the Turtle Posterior Crista

**DOI:** 10.3389/fncel.2017.00356

**Published:** 2017-11-21

**Authors:** Xiaorong Xu Parks, Donatella Contini, Paivi M. Jordan, Joseph C. Holt

**Affiliations:** ^1^Department of Otolaryngology, University of Rochester, Rochester, NY, United States; ^2^Department of Neuroscience, University of Rochester, Rochester, NY, United States; ^3^Department of Pharmacology and Physiology, University of Rochester, Rochester, NY, United States

**Keywords:** hair cell, vestibular, acetylcholine, nicotinic, alpha9, SK

## Abstract

In turtle posterior cristae, cholinergic vestibular efferent neurons (VENs) synapse on type II hair cells, bouton afferents innervating type II hair cells, and afferent calyces innervating type I hair cells. Electrical stimulation of VENs releases acetylcholine (ACh) at these synapses to exert diverse effects on afferent background discharge including rapid inhibition of bouton afferents and excitation of calyx-bearing afferents. Efferent-mediated inhibition is most pronounced in bouton afferents innervating type II hair cells near the torus, but becomes progressively smaller and briefer when moving longitudinally through the crista toward afferents innervating the planum. Sharp-electrode recordings have inferred that efferent-mediated inhibition of bouton afferents requires the sequential activation of alpha9-containing nicotinic ACh receptors (α9^*^nAChRs) and small-conductance, calcium-dependent potassium channels (SK) in type II hair cells. Gradations in the strength of efferent-mediated inhibition across the crista likely reflect variations in α9^*^nAChRs and/or SK activation in type II hair cells from those different regions. However, in turtle cristae, neither inference has been confirmed with direct recordings from type II hair cells. To address these gaps, we performed whole-cell, patch-clamp recordings from type II hair cells within a split-epithelial preparation of the turtle posterior crista. Here, we can easily visualize and record hair cells while maintaining their native location within the neuroepithelium. Consistent with α9^*^nAChR/SK activation, ACh-sensitive currents in type II hair cells were inward at hyperpolarizing potentials but reversed near −90 mV to produce outward currents that typically peaked around −20 mV. ACh-sensitive currents were largest in torus hair cells but absent from hair cells near the planum. In current clamp recordings under zero-current conditions, ACh robustly hyperpolarized type II hair cells. ACh-sensitive responses were reversibly blocked by the α9nAChR antagonists ICS, strychnine, and methyllycaconitine as well as the SK antagonists apamin and UCL1684. Intact efferent terminals in the split-epithelial preparation spontaneously released ACh that also activated α9^*^nAChRs/SK in type II hair cells. These release events were accelerated with high-potassium external solution and all events were blocked by strychnine, ICS, methyllycaconitine, and apamin. These findings provide direct evidence that activation of α9^*^nAChR/SK in turtle type II hair cells underlies efferent-mediated inhibition of bouton afferents.

## Introduction

Vestibular efferent neurons (VENs) branch extensively to produce many presynaptic varicosities abutting hair cells, and both bouton and calyceal afferent processes (Smith and Rasmussen, 1968; Sans and Highstein, 1984; Lysakowski and Goldberg, 1997; Purcell and Perachio, 1997; Jordan et al., 2013). VENs, upon stimulation, may inhibit and/or excite the background discharge of vestibular afferents by activating acetylcholine receptors (AChRs) at each of these synaptic contacts (Goldberg and Fernández, 1980; Rossi et al., 1980; Highstein and Baker, 1985; Brichta and Goldberg, 2000b; Boyle et al., 2009; Mathews et al., 2017). Pharmacological data indicate that the subsequent activation of both nicotinic (nAChRs) and muscarinic ACh receptors (mAChRs) on hair cells and afferents underlie many of these efferent-mediated inhibitory and excitatory responses (Guth et al., 1998; Holt et al., 2011, 2017). That efferent responses differ among hair cells and/or afferents across the neuroepithelium also suggest that these cholinergic receptors are likely differentially distributed.

The morphophysiological organization of hair cells and afferents in the turtle crista has been well-characterized (Brichta and Peterson, 1994; Brichta and Goldberg, 2000a). The turtle posterior crista consists of two saddle-shaped hemicristae that begin medially near the non-sensory torus and broaden as they move laterally toward the planum. Each hemicrista contains both type I and type II vestibular hair cells. While type I hair cells and their accompanying calyx and dimorphic (CD) afferents are restricted to a central zone, type II hair cells and their corresponding bouton afferents are distributed throughout the hemicrista. Bouton afferents are often further differentiated, based on their location along the length of the hemicrista, as either near the torus (BT), near the planum (BP), or innervating middle regions (BM). Not surprisingly, given these various morphological distinctions, both hair cell and afferent physiology vary substantially along the torus-to-planum axis (Brichta and Goldberg, 2000a; Brichta et al., 2002).

Afferent responses to efferent stimulation also differ with respect to afferent class and location (Brichta and Goldberg, 2000b). During efferent stimulation, CD afferents typically exhibit both fast and slow excitatory responses while responses from bouton afferents are more heterogeneous. BT afferents are profoundly inhibited while BP afferents are weakly excited. BM afferents show a mixture of efferent-mediated inhibition and excitation. Electrophysiological and pharmacological data have indicated that efferent-mediated fast and slow excitation of CD afferents is attributed to the activation of α4α6β2-containing nicotinic ACh receptors (α4α6β2^*^nAChRs) and mAChRs, respectively (Holt et al., 2006, 2015a, 2017). These same studies have also inferred that efferent-mediated inhibition of bouton afferents is initiated through the activation of alpha9-containing nicotinic ACh receptors (α9^*^nAChRs) on type II hair cells. Calcium influx following α9^*^nAChR activation presumably opens proximal calcium-dependent, small-conductance, potassium channels (SK) that hyperpolarize the hair cell, decrease neurotransmitter release, and inhibit afferent firing (Holt et al., 2001, 2006; Weisstaub et al., 2002). The role of SK channels in α9^*^nAChR-mediated responses has been repeatedly confirmed in a number of hair cell preparations, including turtle, with blockade by the SK antagonists apamin, scyllatoxin, and dequalinium (Nenov et al., 1996b; Yuhas and Fuchs, 1999; Glowatzki and Fuchs, 2000; Oliver et al., 2000; Holt et al., 2006). Recent work has also demonstrated that BK potassium channels are involved in α9^*^nAChR-mediated responses in outer hair cells from the high frequency regions of the rat cochlea (Wersinger et al., 2010; Rohmann et al., 2015). In turtle, the transition from strong inhibition in BT afferents to weak excitation in BP units suggests that the relative expression of α9^*^nAChRs and/or SK may decrease when moving from hair cells in the torus toward those in the planum. While molecular biological and immunohistochemical data for α9^*^nAChRs in the turtle crista are consistent with this transition (Dailey et al., 2000; Holt et al., 2015a), such inferences have not been tested. Furthermore, a role for BK channels in α9^*^nAChR-mediated responses in turtle vestibular hair cells has also not been explored. Direct recordings from type II hair cells in the turtle crista are needed: (1) To confirm the role of α9^*^nAChRs and SK channels in efferent-mediated inhibition of bouton afferents; and (2) To determine if variations in these efferent receptor mechanisms can account for the differences in efferent-mediated responses among bouton afferents.

Electrophysiological and pharmacological studies have previously been conducted on dissociated vestibular hair cells in various species including turtle (Brichta et al., 2002; Holt et al., 2003; Derbenev et al., 2005; Li and Correia, 2011; Zhou et al., 2013). However, the combined use of harsh mechanical insult and enzymatic treatments utilized in the dissociation of hair cells have led to a number of significant physiological alterations including ion channel function and the disruption of cholinergic signaling mechanisms (Armstrong and Roberts, 1998; Holt et al., 2001; Zhou et al., 2013). Furthermore, the native location of the hair cell being recorded is typically unknown after dissociation. To circumvent many of these issues, a number of intact vestibular organ preparations have been developed (Masetto et al., 1994; Weng and Correia, 1999; Chatlani, 2011; Lim et al., 2011, 2014; Meredith and Rennie, 2015; Contini et al., 2017). In this work, we used whole-cell patch-clamp methodologies to record directly from type II hair cells in a semi-intact, turtle-crista preparation. To confirm if α9^*^nAChRs and SK potassium channels were in fact utilized by crista type II hair cells, we characterized their response to ACh under a panel of pharmacological manipulations including several α9nAChR and SK channel antagonists. Preliminary accounts of this work have appeared in abstract form (Parks et al., 2012).

## Materials and methods

### Acquisition and preparation of vestibular tissue

All experimental procedures involving animals were in accordance with recommendations made by NIH's Guide for the Care and Use of Laboratory Animals and approved by the University Committee for Animal Resources (UCAR) at the University of Rochester. Both male and female red-eared slider turtles (*Trachemys scripta elegans*, 100–400 g, ~7–15 cm carapace length) were deeply anesthetized with sodium pentobarbital. Under deep anesthesia, turtles were decapitated and the head was split along the parasagittal axis. For immunohistochemistry, both halves were transferred to a dissection dish with oxygenated turtle Ringers containing (in mM): 105 NaCl, 4 KCl, 0.8 MgCl_2_, 2 CaCl_2_, 25 NaHCO_3_, 2 Na-pyruvate, 10 glucose, pH 7.2–7.3 (after bubbling with 95% O_2_ / 5% CO_2_ mixture). For hair cell recordings, the resulting two halves were placed in a dissection dish with freshly-prepared, oxygenated external solution containing (in mM): 126 NaCl, 0.5 (or 3) KCl, 2.2 MgCl_2_, 2.8 CaCl_2_, 10 Na-HEPES, 6 D-Glucose, and 2 Na pyruvate, pH 7.6, osmolality ~280 mOsM. The external solution was chilled in an ice bath for dissections while all solutions used during patch-clamp recordings were maintained and applied at room temperature (i.e., 21–25°C). Composition of our high-potassium external solution was identical to our normal external solution above with the exception of an equimolar substitution of NaCl with KCl to elevate potassium concentrations to 40 mM.

### Hair cell recordings

**Figure 1:**
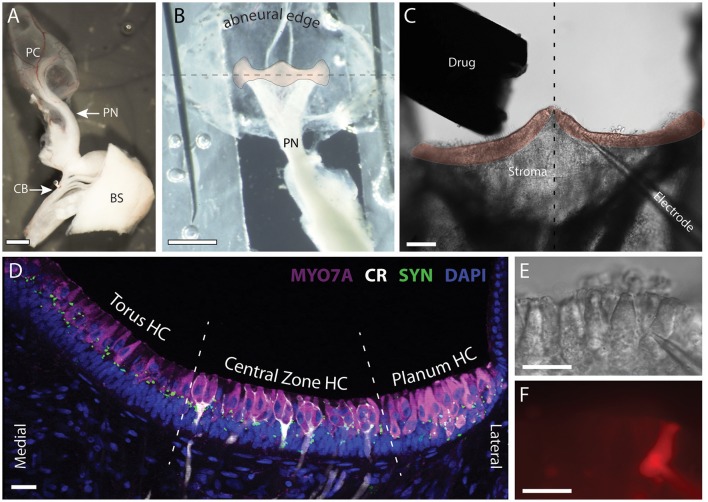
Patch clamp recordings were made from intact hair cells in a split-epithelial preparation. **(A)** The posterior canal (PC), the posterior nerve (PN), and its connectivity to the posterior division of cranial nerve VIII are microdissected from the turtle inner ear. A small section of the brainstem (BS), anterior trunk of CNVIII, and the efferent crossbridge (CB), are also preserved. **(B)** The roof of the posterior canal ampulla is cut, splayed, and immobilized with minutien pins to expose a bird's eye view of the crista neuroepithelium (orange overlay). The abneural edge of the ampulla is folded along the longitudinal torus-to-planum axis of the crista (dashed line) and wrapped underneath to rotate the crista forward into the bottom of the recording dish. **(C)** The upward facing stroma and neuroepithelium were removed by ripping the tissue to expose the underlying hair cells and afferents for subsequent patch-clamp recordings. The drug delivery pipette and nearby patch electrode are also shown. Dashed line bisects the torus to reveal the left and right hemicristae (orange overlay). **(D)** Confocal image projection of a longitudinal section of a turtle hemicrista where hair cells from the torus, central zone (CZ), and planum regions were stained with antibodies to myosin 7A (MYO7A, magenta). Anti-calretinin (CR, white) labeled calyx-bearing afferents in the central zone and type II hair cells in the planum. Synapsin antibodies (SYN, green) labeled vestibular efferent terminals across the neuroepithelium. Orientation of section corresponds to the right hemicrista in **(C)**. Dashed lines delineate the approximate boundaries of torus, central zone, and planum regions from which hair cells (HC) were recorded. **(E,F)** DIC and fluorescent image of patch pipette and type II hair cell near the torus, respectively. Alexa594 sodium hydrazide (50 μM) was included in the patch pipette. Scale Bars (in μm): **(A,B)** = 500; **(C)** = 100; **(D–F)** = 20.

The semi-intact, turtle canal, split-epithelial preparation allowed for direct access to type II hair cells for patch-clamp recordings, while maintaining each cell's native location and avoiding the use of harsher mechanical and enzymatic dissociation conditions normally associated with many isolated hair cell preparations. Details regarding the split epithelial crista preparation have been previously described (Chatlani, 2011; Jordan et al., 2013; Contini et al., 2017). Most of our recordings specifically utilized the posterior canal crista (Figure 1) which could be microdissected from the turtle's inner ear while retaining most of its innervation and connection to a small section of the brainstem (BS). The reduced crista preparation was then transferred to our recording chamber. To gain access to the neuroepithelium, the membranous wall of the ampulla above the crista was cut and splayed. The abneural edge was folded underneath along the longitudinal axis of the crista and pinned onto a layer of parafilm which rotated the neuroepithelium toward the bottom of the dish (Figures 1B,C). A tungsten probe was then used to score several furrows in the upward facing stroma. Fine forceps were then used to clasp edges of the newly created stromal flaps followed by a quick lateral motion to rip and remove the upper layers to expose the underlying hair cells for subsequent patch-clamp recordings (Figure 1C). The anterior crista was occasionally prepared for recordings in a similar way. The split preparation and associated hair cells were viewed using a Zeiss AxioExaminer microscope (Zeiss, Germany) equipped with differential interference contrast optics, 10x and 63x water-immersion objectives, and an optovar module in the reflector turret with 1.0x, 1.6x, and 2.5x additional magnification. Whole-cell patch-clamp recordings were performed using borosilicate electrodes pulled on a Sutter P97 micropipette puller. Patch electrodes, with impedances ranging 1.4–8.8 MΩ (mean = 4.5 ± 0.09), were backfilled with internal solution (in mM: 130 KCl, 2 MgATP, 0.1 CaCl_2_, 10 Na-HEPES, 11 EGTA, pH 7.4). Alexa Fluor® 594 hydrazide (Invitrogen/Molecular Probes, Grand Island, NY), at a concentration of 50 μM, was included in the internal solution to provide visual confirmation of the recorded cell. Liquid junction potentials and the capacitance of the electrode were nulled prior to sealing. After forming a seal and going whole cell, up to 60% of the series resistance was compensated using amplifier controls. Voltage and current clamp protocols were constructed and applied using Clampex10 (Molecular Devices, Sunnyvale, CA). Data were sampled at 20 K, filtered at 5 K, and collected using an Axopatch 200B/Digidata 1440 system. Current rundown was observed in some hair cells but was limited to <10% of total initial amplitudes after 20 min of recording.

### Drugs

Most of our chemical agents and drugs including acetylcholine (ACh), tropisetron (ICS), strychnine (STR), and apamin (APA) were purchased from Sigma Aldrich (St. Louis, MO). The drugs methyllycaconitine (MLA) and UCL1684 (UCL) were acquired from Tocris Bioscience (Minneapolis, MN). Each drug was prepared first as a concentrated stock solution in ultra-filtered water and stored as frozen aliquots. On experimental days, aliquots were removed and thawed after which an appropriate volume was added to external solution to arrive at a desired working concentration. Both external and daily working drug solutions were loaded into 10-ml reservoirs supplying a gravity-fed multidrug pipette whose single tip was positioned 300–500 μM from the patched cell using a micromanipulator (Figure 1C). Solutions were maintained at room temperature and each reservoir was continuously oxygenated during patch-clamp experiments. During each hair cell recording, external solution was first superfused to ensure absence of mechanical artifacts before delivering ACh. ACh was then typically applied to the cell for 5–20 s to elicit a current or voltage response during voltage clamp or current clamp protocols, respectively. This time frame was chosen in order to ensure that we were observing the maximal effects of ACh. To assess the current-voltage (I–V) relationship of ACh-mediated responses in type II hair cells under voltage clamp, we examined the effect of ACh on the total currents produced by both voltage step and ramp protocols. During the voltage step series, type II hair cells were held at −67 mV for 56 ms, followed by a step to −137 mV for 250 ms, and then back to −67 mV for ~100 ms. This was repeated after incrementing the step potential by 10 mV each time until we reached a final step voltage of 43 mV. Alternatively, during the ramp protocol, type II hair cells were also held at −67 mV, stepped to −130 mV for 100 ms, and then ramped to 40 mV over 400 ms, followed by returning to a holding potential of −67 mV. The ramp was set to repeat continuously until stopped by the user. In either case, the ACh-sensitive current was isolated by subtracting the current obtained under control conditions from those generated during ACh exposure. For subsequent pharmacological challenge of ACh responses, we superfused each cholinergic antagonist for at least 30 s before probing the same cell with the co-application of ACh and that particular antagonist. Reversibility was demonstrated for all antagonists with the exception of apamin, whose large molecular weight and high-binding affinity (Stocker, 2004) presumably impeded its clearance from neuroepithelium. Prolonged effects with apamin were previously documented in sharp-electrode recordings from the turtle posterior crista (Holt et al., 2006).

### Immunohistochemical (IHC) studies

As previously reported (Jordan et al., 2015), the vestibular labyrinth was extracted from the temporal bone and drop-fixed in freshly prepared 4% paraformaldehyde (PFA, Electron Microscopy Services, Hatfield, PA) for 4–6 h. Canal cristae were then dissected in 0.1 M phosphate buffer, and transferred to 30% sucrose (in 0.1 M PB) for at least 1 h at 4°C. Cryoprotected cristae were then embedded in 12% gelatin (prepared in 30% sucrose) and chilled at 4°C until firmly solidified. The resulting gelatin block was frozen on the stage of a sliding microtome where 40-μm longitudinal sections were cut and placed into a collection vial. Gelatin was removed by warming the vial containing the crista sections followed by washing and resuspension in 0.1 M PB. Crista sections were blocked in 0.5% Triton X–0.1 M PB containing 5% normal donkey serum (Jackson ImmunoResearch, Westgrove, PA). Tissue sections were then incubated overnight with antibodies against calretinin (Millipore, Billerica, MA; item #AB5054, lot LV1552190, 1:1,000 dilution), Myosin VIIA (DSHB item 138-1C, 1:100 dilution), and synapsin (Millipore Cat# AB1543 RRID:AB_2200400). Calretinin was primarily used to label calyx-bearing afferents in the central zone of the crista. Myosin VIIA was used to label all hair cells, and synapsin was used to demarcate turtle efferent varicosities (Jordan et al., 2015). Following multiple washes in 0.1 M PB, sections were incubated in the dark for 2 h at room temperature with Alexa Fluor®-conjugated secondary antibodies (Invitrogen/Molecular Probes, Grand Island, NY) at 1:500 dilutions in 0.1 M PB. Sections were first washed with 0.1 M PB, reacted with DAPI (4′,6′-diamidino-2-phenylindole, 1:1,000 of 1 mg/ml solution, Sigma, St. Louis, MO) for 5 min, and rinsed with distilled H_2_O for 5 min. An eyelash probe was used to transfer tissue sections to Plus Slides (Fisher Scientific, Pittsburgh, PA) where they were cover-slipped with SlowFade Gold (Invitrogen, Grand Island, NY). Confocal images were captured with Olympus Fluoview software suite on an Olympus FV1000 laser scanning confocal microscope (Olympus America, Center Valley, PA) in the URMC Confocal & Conventional Microscopy Core. Captured images were exported as TIFF files and prepared as figures using Adobe Photoshop and Illustrator (Adobe, Inc., San Jose, CA).

### Data analysis

Data were analyzed and plotted using Clampfit (Molecular Devices, Sunnyvale, CA), Excel (Microsoft, Redmond, WA) and Igor Pro 6.37 (Lake Oswego, OR). All statistical analyses were done in GraphPad Prism (GraphPad Software, La Jolla, CA). Unless otherwise stated, values are expressed as means ± SEM. Differences in hair cell responses to ACh with and without pharmacological antagonists were evaluated with paired *t*-tests. Unpaired *t*-tests were used to evaluate differences in ACh-mediated responses between hair cells from the torus and central zone regions. For plotting the dose-response relationship for ACh, hair cell responses to different concentrations of ACh were normalized relative to the cell's response to 300 μM ACh. Normalized responses at each concentration were then averaged across multiple hair cells and the resulting means were plotted and iteratively fitted with the Hill equation:

$$R_{X}=R_{0}+(R_{\infty }-R_{0})/(1+{\left(X_{0.5}/X\right)}^{h})$$

where *X* is the concentration of ACh, *X*_0.5_ is the concentration of ACh that produces 50% of the maximal response, *R*_*X*_ is the response to ACh at concentration *X, R*_0_ is the lower asymptote or response when ACh dose = 0, *R*_∞_ is the upper asymptote at an infinite ACh concentration, and *h* is the Hill coefficient.

## Results

For orientation, the cellular organization of the neuroepithelium in our split-epithelial preparation is best illustrated using an immunohistochemical image taken from longitudinal sections of the posterior crista (Figure 1D). Here, hair cells, calyx-bearing afferents, and efferent terminals are stained with myosin 7A (magenta), calretinin (white), and synapsin (green), respectively. Type II hair cells and efferent terminals are distributed throughout the crista while type I hair cells are confined to the central zone (CZ). Type I hair cells in the CZ are distinguished by the presence of calyx-bearing afferents which can be easily visualized during patch-clamp recordings using DIC optics. For this study, we exclusively recorded from type II hair cells located in one of three regions of the crista designated as Torus, Central Zone, or Planum (Figure 1D). The bulk of the recordings were made in type II hair cells from the torus region. All type II hair cells were identified by their crista location, characteristic shape, and lack of calyx ending, all of which was confirmed in many recordings by visualizing fluorescent fills with Alexa594-hydrazide after going whole cell (Figures 1E,F). During patch-clamp recordings, the lack of the signature type I hair cell potassium current IKL provided further confirmation that we were recording from type II hair cells (Rennie and Correia, 1994; Rüsch and Eatock, 1996; Brichta et al., 2002). A total of 240 cristae from 165 turtles were collected for this study from which 323 type II hair cells from the three regions were recorded. Cells were deemed healthy provided the cell membrane appeared intact, there was no obvious swelling, and the resting membrane potential was stable at −40 mV or lower.

### Typical recordings from type II hair cells near the torus: acetylcholine-sensitive inward and outward current in type II hair cells

**Figure 2:**
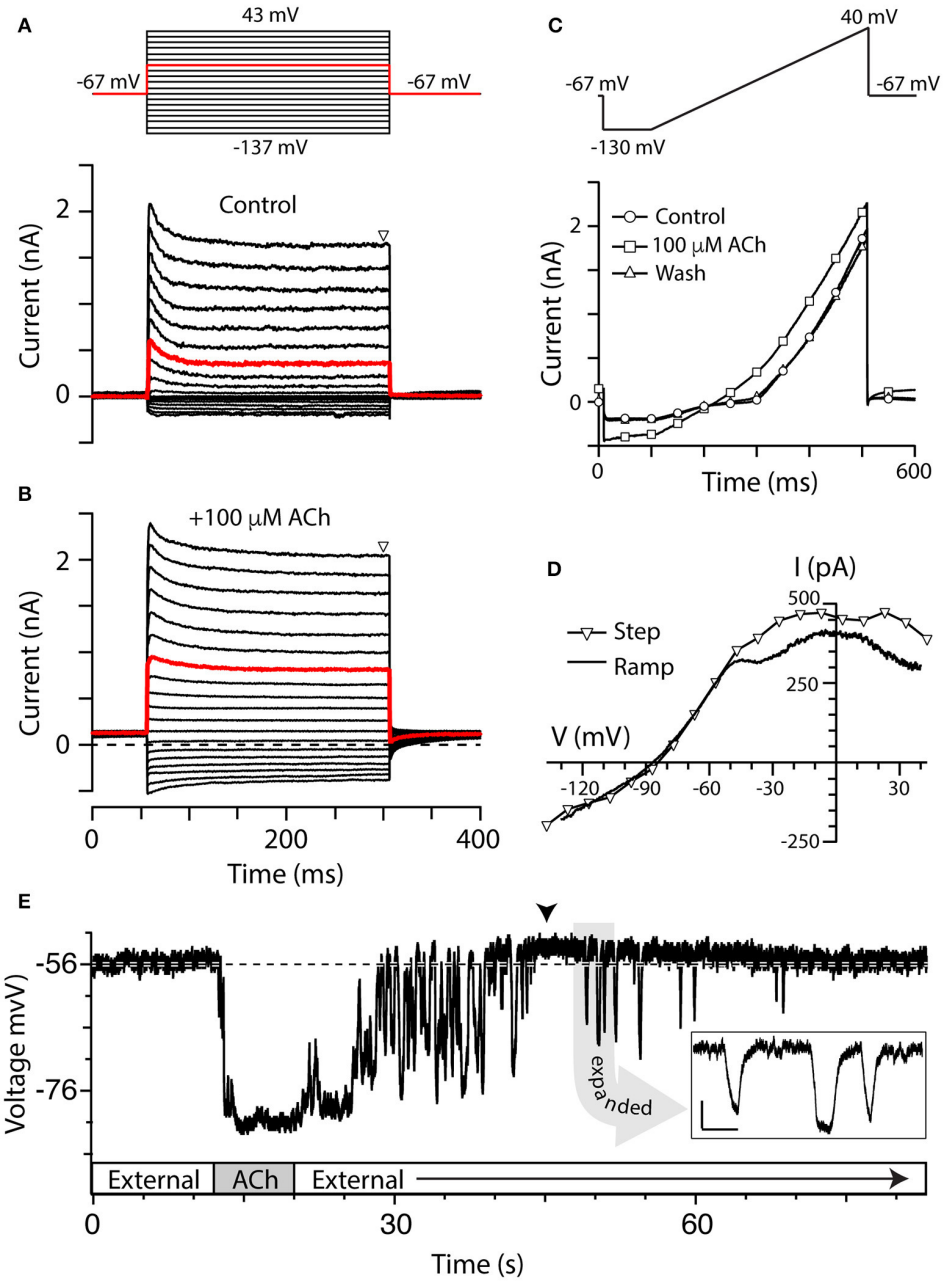
Torus type II hair cells respond to ACh. **(A,B)** Family of current responses in a type II hair cell near the torus before **(A)** and 10 s after the application of 100 μM ACh **(B)**. The cell was first held at −67 mV for 56 ms, then stepped to a voltage from −137 to 43 mV in 10 mV intervals for 250 ms, and then back to −67 mV for 100 ms (protocol at top). Steady state current amplitudes were measured at *t* = 300 ms (triangle). Red traces indicate currents elicited by a voltage step to −17 mV. Dashed line in **(B)** is zero-current level. **(C)** Current recorded from the same cell during a ramp protocol, where the cell was first held at −67 mV, stepped to −130 mV for 100 ms, and then ramped to 40 mV over 400 ms, under control condition (circles), application of 100 μM ACh (squares), or after wash (triangles). **(D)** Current–voltage (I–V) relationship of ACh-sensitive current during voltage step protocol (triangles) was compared to ACh-sensitive currents generated during the ramp protocol (solid line). When voltage steps were used, the ACh-sensitive current was generated by subtracting control current (**A**, lower panel) from the ACh current **(B)** at steady-state. When ramp protocol was used, ACh-sensitive current was generated by subtracting the control current from ACh current. In order to plot the I–V relationship, time points were linearly converted to voltage by multiplying by the ramp speed. **(E)** Gap-free current clamp recording under zero-current conditions were acquired from a torus type II hair cell during the superfusion of external solution and 100 μM ACh. Segments of application indicated in boxes along the time axis. Arrowhead indicates a post-hyperpolarization depolarization. Dashed line demarcates the mean pre-ACh zero-current membrane potential. Inset: Several hyperpolarizing fluctuations observed during post-ACh washout are shown on an expanded time scale (scale bars: 500 ms, 5 mV).

To optimize conditions for observing α9^*^nAChR-mediated responses in turtle posterior crista hair cells, we first recorded the current response of torus type II hair cells near −20 mV before and during the application of 100 μM acetylcholine (ACh). This approach was used since: (1) Bouton afferents innervating type II hair cells near the torus (BT) showed the most robust inhibitory responses during efferent stimulation (Brichta and Goldberg, 2000b; Holt et al., 2006, 2015a); and (2) Peak α9nAChR-mediated activation of SK potassium currents in other hair cell systems ranges from −40 to −10 mV (Fuchs and Murrow, 1992; Nenov et al., 1996a; Glowatzki and Fuchs, 2000; Holt et al., 2003; Gómez-Casati et al., 2005; Li and Correia, 2011). In our initial experiments, the cell was first held at −67 mV for 56 ms, followed by a 250-ms step to −17 mV, then back to −67 mV for an additional 100 ms (exemplified by red traces, Figures 2A,B). Similar voltage step protocols have been used to characterize α9^*^nAChRs in frog vestibular hair cells (Holt et al., 2001, 2003). Under these conditions, the local delivery of 100 μM ACh to torus type II hair cells consistently resulted in an increase in outward current at both the holding and step potential. As would be consistent with the activation of a potassium conductance, where less current is available as one moves toward the reversal potential for potassium (i.e., ~ −95 mV in our experiments), the net current change seen with ACh in Figure 2B was smaller at −67 mV than the net current change observed at −17 mV (i.e., 125 vs. 400 pA).

We examined the effects of ACh on type II hair cell currents using a series of voltage steps or ramp protocol (see Materials and Methods). The step protocol was first performed under control conditions during superfusion of external solution (Figure 2A) and then repeated in the presence of 100 μM ACh (Figure 2B). Consistent with voltage-clamp data from isolated turtle hair cells (Brichta et al., 2002), torus type II hair cells in the split-epithelial preparation typically showed outward currents with relatively fast activation and inactivation kinetics during the 250-ms step, particularly at more depolarizing potentials (Figure 2A). Small inward currents were also observed at hyperpolarizing potentials in most torus type II hair cells. In the presence of 100 μM ACh, the total outward and inward currents increased in amplitude (Figure 2B). With the voltage step protocol, there were a total of 19 voltage sweeps with each sweep requiring 400 ms such that a complete run of the protocol required ~8 s. With the addition of up to 10 s for observing maximal ACh effects, each trial would ideally require on the order of 20 s to record the effects of ACh on current responses at each of the voltage steps. To shorten ACh exposure times and minimize any desensitization that might occur as a result of it, we utilized a ramp protocol (Figure 2C). As with the step protocol, 100 μM ACh was applied for ~5–10 s before reaching the maximal effect. The ramp protocol differed from the step protocol in several ways: (1) It significantly reduced the exposure times to ACh by 50–75%; and (2) a complete set of currents could be captured during the transitions from superfusion of external solution to ACh and back to external solution. As with voltage steps, application of 100 μM ACh during the ramp protocol also increased both outward and inward currents (Figure 2C, squares) as compared to control currents (Figure 2C, circles). The current response to the ramp protocol during wash with external solution was comparable to control currents (Figure 2C, triangles). Although we did not specifically study desensitization in this study, there were no obvious declines in the amplitude of ACh-sensitive currents with sustained ACh application during the ramp protocol.

Figure 2D finally compares the I–V plots of the ACh-sensitive currents generated by the two voltage-clamp protocols. With voltage steps, the I–V plot of the ACh-sensitive current was generated by subtracting control currents during steady-state at *t* = 300 ms (Figure 2A, triangles) from corresponding currents recorded in the presence of 100 μM ACh (Figure 2B, triangles). Those points are plotted as triangles in Figure 2D. ACh-sensitive currents, generated during the 400-ms ramp segment (solid line, Figure 2D), were calculated by subtracting control currents from the total currents generated during the application of 100 μM ACh (Figure 2C). Although the current amplitude was a little reduced with the ramp protocol, the resulting I–V plots of the ACh-sensitive currents generated with either protocol were very similar in shape, and in the estimates of a reversal potential of ~ −86 mV (Figure 2D). Peak ACh-sensitive outward currents typically plateaued around −20 mV and then decreased in amplitude at more depolarized potentials, presumably as a function of a decrease in the driving force for calcium through α9^*^nAChRs. It is worth noting that there was some variability in the shape of the I–V plots across our recording population regarding these two features, but the bases for these differences were not explored in this study. These I–V plots are consistent with the activation of α9^*^nAChRs and SK potassium channels reported in other hair cell systems (Nenov et al., 1996b; Glowatzki and Fuchs, 2000; Holt et al., 2003; Kong et al., 2005). Because the ramp protocol reduced the duration of ACh application while producing similar I–V plots, it became the preferred protocol for comparing I–V relationships in type II hair cells under different pharmacological treatments and at different crista locations.

At hair cell membrane potentials more depolarized than the reversal potential for potassium, the above I–V relationship predicts that the application of ACh should hyperpolarize torus type II hair cells. Current-clamp recordings from torus type II hair cells were used to characterize the effects of ACh on membrane potential. Under zero-current conditions, the membrane potential of a torus type II hair cell shown in Figure 2E was −56 mV which remained stable for over 10 s prior to the application of ACh. An 8-s application of 100 μM ACh hyperpolarized the zero-current potential by ~25 mV which was maintained until ACh was terminated and external solution was returned. We recorded from a total of 290 torus type II hair cells, of which 250 cells responded to 100 μM ACh. The mean ACh-sensitive current at −20 mV was 363 ± 21 pA (*n* = 203). The average zero-current potential (Vz) for responding torus type II hair cells was −51 ± 0.6 mV (*n* = 188) and the average ACh-sensitive hyperpolarization was −25.2 ± 0.7 mV (*n* = 155). There were no obvious differences in voltage-dependent currents or Vz between these 250 cells and the 40 cells that did not respond to 100 μM ACh.

Two other prominent ACh-sensitive voltage changes are worth mentioning. One, in many of our current-clamp recordings, including the example shown in Figure 2E, the termination of ACh application and washout period were associated with a number of large hyperpolarizing fluctuations. The shapes of the events were difficult to characterize during much of the return to baseline given their apparent increased frequency, but a few isolated events could often be seen at later time points (see Figure 2E inset). We initially thought they might be synaptic events associated with the release of ACh from intact efferent terminals. But these fluctuations were not evident in our voltage-clamp recordings, and their wave forms, particularly the rise-time/decay kinetics and protracted peaks, were not well-matched with the general shape of efferent-mediated synaptic events identified in other hair cell recordings (Glowatzki and Fuchs, 2000; Oliver et al., 2000). Furthermore, events of similar magnitude could often have different shapes. There was no obvious periodicity in the fluctuations from hair cell to hair cell where they ranged from being prominent and protracted in some and absent in others. However, because the amplitude and frequency decreased as a function of washout time, we reasoned that these events likely represented α9^*^nAChRs on the hair cell continuing to respond to transient re-exposure to ACh during the washout process. Such fluctuations are not seen in BT/BM afferent recordings during efferent stimulation which suggests that it is likely an artifact of the way in which we apply ACh. Unlike isolated hair cells, the removal of ACh from the synaptic cleft in our preparation during wash would be constrained by the surrounding neuroepithelial tissue which likely varied from cell to cell. The second phenomenon often observed in our current-clamp recordings was that the ACh-induced hyperpolarization was often followed by a depolarization (arrowhead, Figure 2E) which averaged 5.6 ± 1.4 mV (*n* = 56) and lasted for tens of seconds. This depolarization may utilize the same mechanism driving the post-inhibitory excitation (PIE) seen in sharp electrode recordings from turtle bouton afferents during efferent stimulation (Brichta and Goldberg, 2000b; Holt et al., 2006, 2015a).

### Establishing a dose response curve for acetylcholine

**Figure 3:**
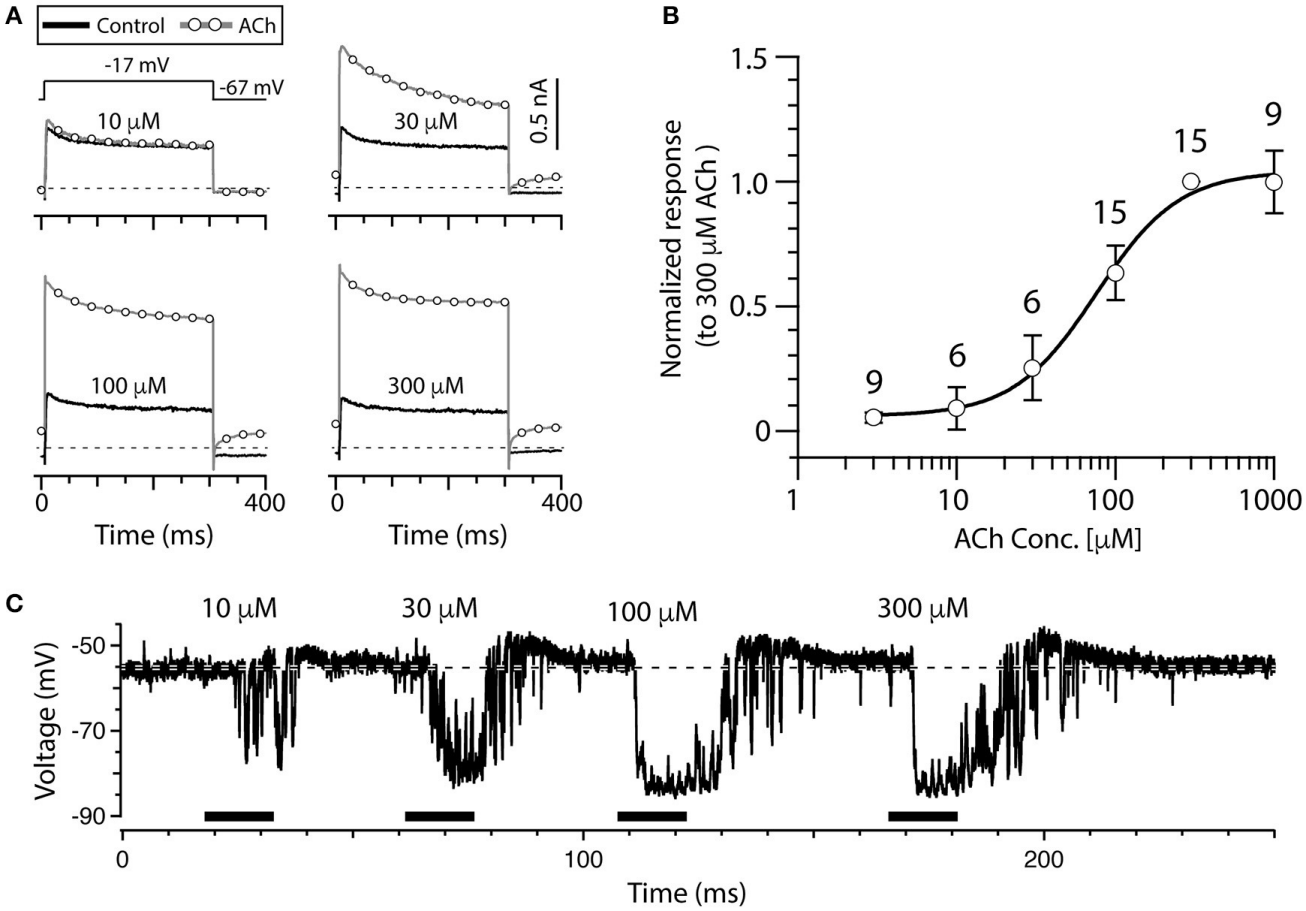
ACh dose-dependently increases outward current in torus type II hair cells. **(A)** Outward currents from a torus type II hair cell during a voltage step to −17 mV from a holding potential of −67 mV, during superfusion of external solution (solid line) or the application of 10, 30, 100, and 300 μM ACh (circles). Step protocol in top of first panel applies to all traces. Dotted line represents zero-current level. **(B)** The amplitude of ACh-sensitive current responses to 3, 10, 30, 100, 300, and 1,000 μM ACh were all normalized to responses to 300 μM ACh. Error bars reflect SEM. Solid line is a Hill Equation fit of the data with EC_50_ of 76 μM. Number of cells indicated above symbols. **(C)** Gap-free current clamp recording under zero-current conditions were acquired from a torus type II hair cell during the superfusion of increasing concentrations of ACh (horizontal black bars). Each indicated concentration was applied for 15 s. Hair cells were superfused with external solution in the segments before and after ACh application. Dashed line demarcates the mean baseline zero-current membrane potential.

The data presented thus far has demonstrated the response of type II hair cells to only 100 μM ACh, but we wanted to further assess the sensitivity to ACh by characterizing a complete dose response curve in the split-epithelial preparation (Figure 3). To optimize conditions for this analysis, the current responses of torus type II hair cells to different ACh concentrations were recorded during a simple voltage step protocol. The protocol consisted of repeated single voltage steps from a holding potential of −67 to −17 mV for 300 ms, followed by a return to a holding potential of −67 mV for 100 ms. The four panels in Figure 3A show the current responses of a single torus type II hair cell to this voltage step during the superfusion of external solution (black, solid lines) and increasing ACh concentrations (gray, open circles). This particular cell exhibited a small response to 10 μM ACh which continued to grow in amplitude up to 300 μM ACh. The ACh-sensitive current was calculated by subtracting the corresponding control current from the total currents generated in response to each concentration of ACh. In each group and for each cell, the peak amplitude of the ACh-sensitive current to different ACh concentrations was always normalized to the peak amplitude of the ACh-sensitive current response to 300 μM ACh (Note the lack of an error bar at this dose). Mean normalized response amplitudes were then plotted vs. the ACh concentration (open circles, Figure 3B). Mean data were fitted with a Hill Equation (Figure 3B, solid line) that identified an EC_50_ of 76 μM for ACh in our preparation. Similar observations were also seen in current-clamp recordings where increasing ACh concentrations produced larger and longer hyperpolarizations (Figure 3C). In this example, the depolarization following each hyperpolarization also grew in size and duration. Interestingly, the response to 10 μM ACh (Figure 3C) primarily consisted of a series of transient hyperpolarizations that resembled the fluctuations seen on washout of higher ACh concentrations (Figure 2E). This provides additional support that these fluctuations likely represent continued activation of α9^*^nAChRs and SK channels as the levels of exogenous ACh concentrations fall during the washout process.

### α9nAChR antagonists blocked ACh-sensitive current in type II hair cells

**Figure 4:**
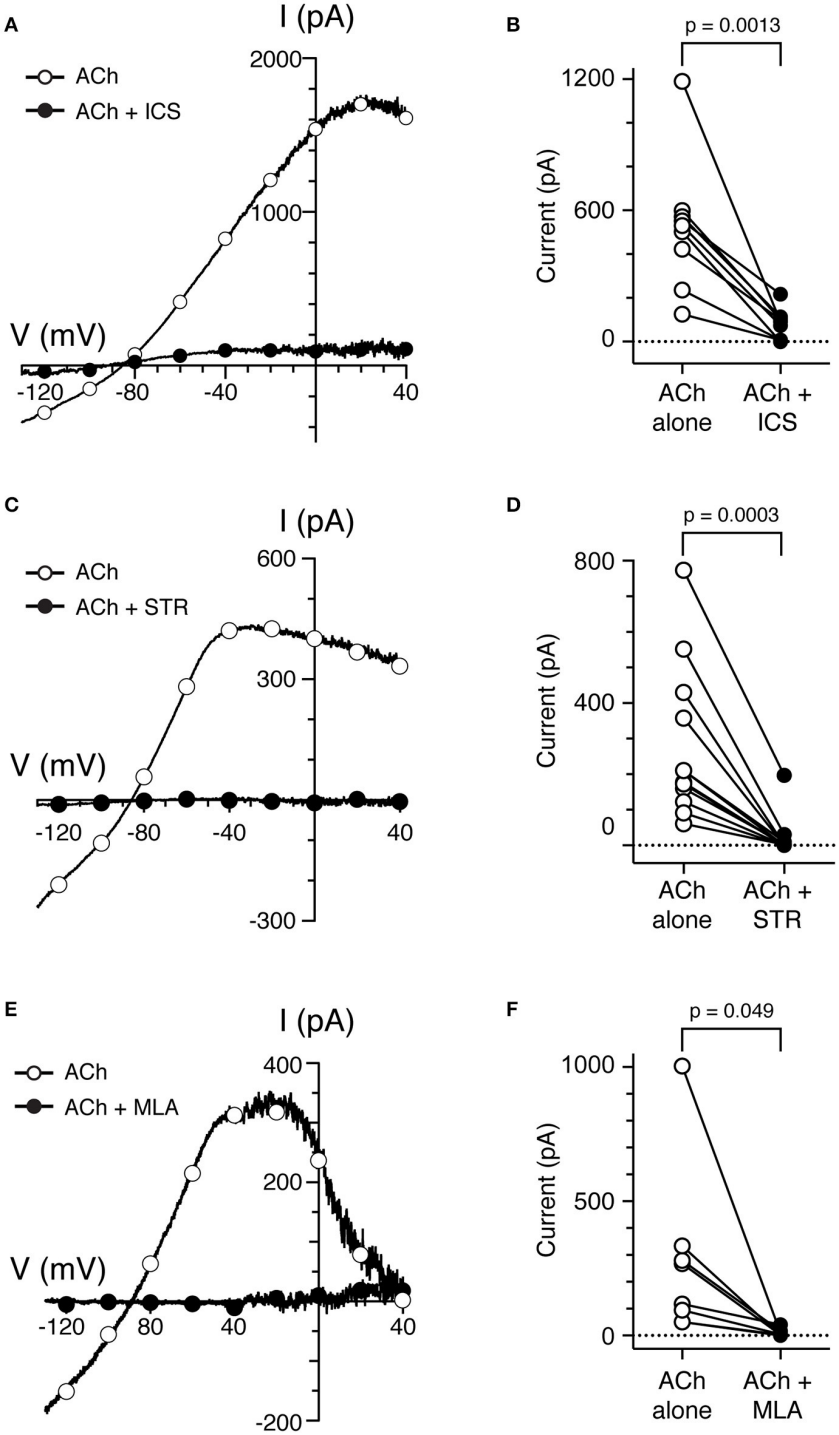
Cholinergic antagonists of α9*nAChRs block ACh-sensitive currents in torus type II hair cells. **(A,C,E)** ACh-sensitive currents in torus type II hair cells acquired with the ramp protocol during the application of 100 μM ACh alone (open circles) or 100 μM ACh with 5 μM ICS **(A)**, 3 μM STR **(C)**, or 200 nM MLA **(E)**. Currents in the presence of antagonists shown as filled circles. **(B,D,F)** Peak ACh-sensitive current at −20 mV in type II hair cells are plotted before (ACh, open circles) and after ICS, STR, or MLA (filled symbols), respectively.

To determine if α9^*^nAChRs were mediating the responses of torus type II hair cells to ACh, several well-characterized cholinergic antagonists known to block α9^*^nAChRs were utilized (Elgoyhen et al., 1994, 2001; Rothlin et al., 1999; Verbitsky et al., 2000). In sharp-electrode recordings (Holt et al., 2006, 2015a), efferent-mediated inhibition of turtle BT afferents was blocked by the α9^*^nAChR antagonists ICS, strychnine (STR), and methyllycaconitine (MLA). In whole-cell, patch-clamp recordings, we tested the effects of these three different cholinergic compounds on the responses to ACh. We recorded the current responses of torus type II hair cells to the ramp protocol before and during application of 100 μM ACh, with or without the addition of these cholinergic antagonists. To isolate ACh-sensitive currents, the control currents during the superfusion of external solution or nAChR antagonists alone were subtracted from the currents generated during the application of ACh, or ACh plus each nAChR antagonist, respectively. The time axis of the 400-ms ramp segments of ACh-sensitive current during ACh alone and ACh plus nAChR antagonist were subsequently transformed to voltage as a function of the known ramp speed. The resulting I–V curve for a torus type II hair cell during 5 μM ICS is shown in Figure 4A. In this example, ICS blocked ~91% of ACh-sensitive current when measuring current amplitudes at −20 mV. The ACh-sensitive current returned to control levels after washout of ICS (data not shown). In nine cells, 85% of the ACh-sensitive current at −20 mV was blocked by 3–10 μM ICS (525.5 ± 98.9 vs. 78.6 ± 22.9 nA, *p* = 0.0013, Figure 4B). As shown in Figure 4C, the ACh-sensitive current in another torus type II hair cell (open circles) was also potently blocked by 3 μM STR (filled circles). In 12 cells, 93% of the ACh-sensitive current at −20 mV was significantly blocked by 1–3 μM STR (276.3 ± 61.6 vs. 20.4 ± 16.2 nA, *p* = 0.0003, Figure 4D). Finally, in Figure 4E, the ACh-sensitive current in a type II hair cell (open circles), measured at −20 mV, was completely abolished by 200 nM MLA (filled circles). The percentage of ACh-sensitive current significantly blocked by 100–300 nM MLA in eight cells was 96% (278.3 ± 123.2 vs. 12.2 ± 6.5 nA, *p* = 0.049, Figure 4F). The ACh-sensitive current was also reversibly blocked by 1 μM αBTX in one torus type II hair cell (data not shown). The finding that four α9nAChR antagonists (ICS, STR, MLA, and αBTX) blocked ACh-sensitive currents in torus type II hair cells is consistent with the activation of α9nAChRs and with the reported pharmacology of efferent-mediated inhibition in sharp electrode recordings (Holt et al., 2006, 2015a).

### SK channel antagonists blocked ACh-sensitive outward current and hyperpolarization of type II hair cells

**Figure 5:**
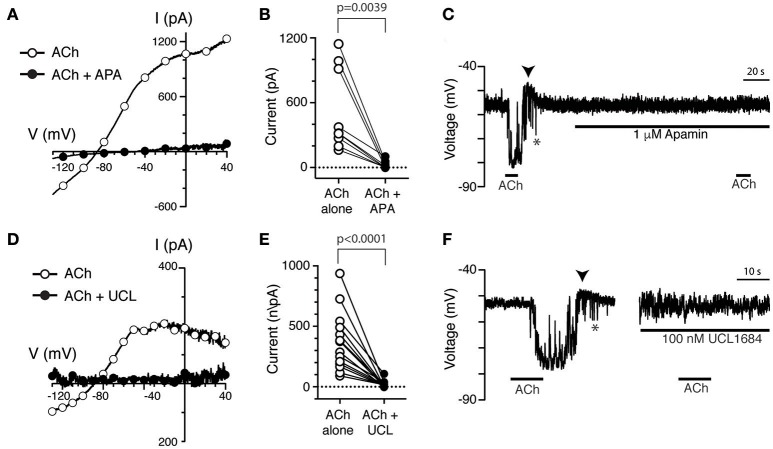
The selective SK channel antagonists apamin and UCL1684 block ACh-sensitive currents in torus type II hair cells. **(A,D)** ACh-sensitive currents in torus type II hair cells acquired with the ramp protocol during the application of 100 μM ACh alone (open circles) or in the presence of 100 μM ACh with 500 nM apamin **(A)** or 100 nM UCL1684 **(D)**. Currents in the presence of antagonists shown as filled circles. **(B,E)** Peak ACh-sensitive current at −20 mV in different type II hair cells are plotted before (ACh, open circles) and after apamin (APA) or UCL1684 (filled circles), respectively. **(C,F)** ACh-sensitive voltage changes recorded under zero-current, current-clamp conditions during the application of 100 μM ACh alone or 100 μM ACh with 1 μM apamin **(C)** or 100 nM UCL1684 **(F)**. Arrowheads indicate post-hyperpolarization depolarization. Gap between traces in **(F)** represents segment of activity removed to allow side by side comparison.

Previous sharp-electrode recordings have shown that small-conductance, calcium-dependent potassium channels (SK) are involved in efferent-mediated inhibition of bouton afferents in the turtle posterior crista (Holt et al., 2006). Here, a role for SK channels in the response of type II hair cells to ACh was probed using the selective SK channel blocker apamin (Köhler et al., 1996; Ishii et al., 1997). Compared to ACh alone (Figure 5A, open circles), a torus type II hair cell displayed little ACh-sensitive current after the application of 500 nM apamin (Figure 5A, filled circles). In nine torus type II hair cells, 0.5–1 μM apamin significantly blocked 95% of the ACh-sensitive current at −20 mV (520.2 ± 126.8 vs. 23.0 ± 11.8, *p* = 0.0039, Figure 5B). Similarly, in current clamp mode under zero-current conditions, the robust hyperpolarization of type II torus hair cells during the application of 100 μM ACh was also completely inhibited during the application of 1 μM apamin (Figure 5C). Similar observations were made in 13 type II hair cells tested under zero-current conditions in current-clamp mode. In 4 of these 13 type II hair cells, a small depolarization was unmasked following apamin treatment (data not shown), but was not pharmacologically challenged to identify if it was attributed to the activation of α9^*^nAChRs.

We also investigated the effects of the non-peptide SK channel blocker, UCL1684, on ACh-sensitive currents in torus type II hair cells. Like apamin, UCL1684 can also block SK channels in low nanomolar concentrations (Rosa et al., 1998; Weatherall et al., 2011). However, we found no reports that UCL1684 had been used to challenge SK channels in hair cell preparations. In voltage-clamp experiments, the ACh-sensitive current in a torus type II hair cell (Figure 5D, open circles) was almost completely blocked by 100 nM UCL1684 (Figure 5D, filled circles). In 14 torus type II hair cells, 95% of the ACh-sensitive current was significantly blocked by UCL1684 (370.8 ± 64.7 vs. 20.1 ± 7.5, *p* < 0.0001, Figure 5E). Similar to apamin, UCL1684 also blocked the ACh-sensitive hyperpolarization (Figure 5F) under zero-current current-clamp conditions, which returned after UCL1684 was washed off (data not shown). Note that the depolarization (arrowheads) that followed the ACh-mediated hyperpolarization was also blocked by both apamin (Figure 5C) and UCL1684 (Figure 5F).

Finally, in other hair cell preparations, it has been shown that ACh can activate both SK and BK potassium channels on the basis of their differential sensitivity to apamin and the selective BK blocker iberiotoxin (IBTX), respectively (Kong et al., 2005; Wersinger et al., 2010; Zhou et al., 2013; Rohmann et al., 2015). However, given that most of the ACh-sensitive outward current was blocked by apamin and UCL1684, BK potassium channels likely do not play a significant, if any, role. This was demonstrated by challenging α9^*^nAChR-mediated responses with 100 nM IBTX in turtle type II hair cells. The peak amplitude of ACh-sensitive currents generated using the ramp protocol were not significantly different before and after IBTX (193.0 ± 65.4 vs. 176.3 ± 61.5, *p* = 0.3813, *n* = 4). Furthermore, IBTX failed to alter the amplitude or time course of ACh-sensitive hyperpolarization in current-clamp recordings in two other type II hair cells (Data not shown). Collectively, these observations indicate that SK channels are primarily responsible for ACh-sensitive currents and hyperpolarization in torus type II hair cells. That the hyperpolarizing fluctuations (Figures 5C,F, asterisks) and post-hyperpolarization depolarization (Figures 5C,F, arrowheads) were also blocked by apamin and UCL suggest that they too require SK-mediated hyperpolarization.

### Comparison of type II hair cells at different regions

**Figure 6:**
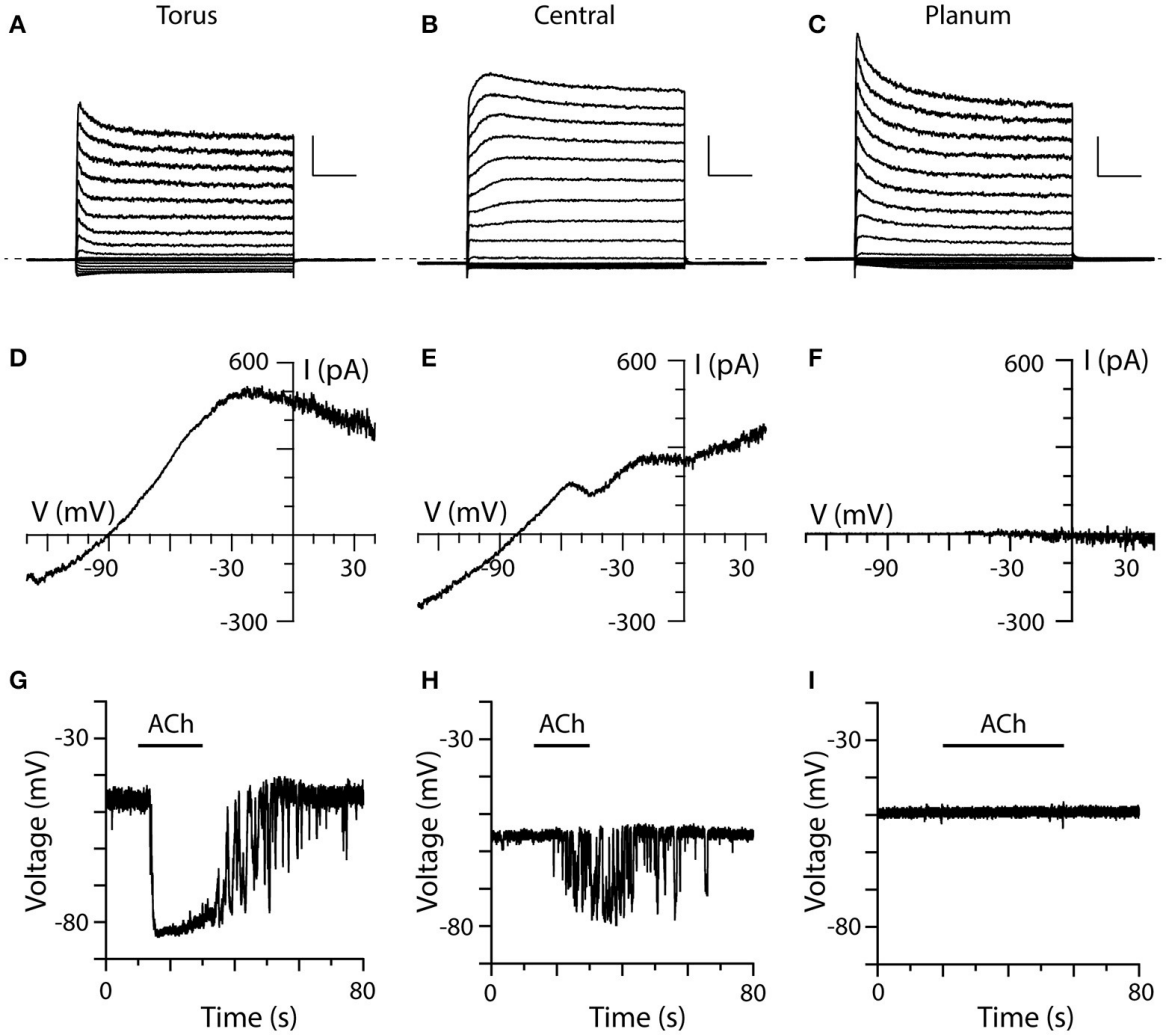
Comparison of ACh-sensitive currents in type II hair cells from three different regions of crista. **(A–C)** Typical current recordings to voltage steps from type II hair cells near the torus, within the central zone, and near the planum, respectively. Each cell was first held at −67 mV for 56 ms, then stepped to a voltage from −137 to 43 mV at an interval of 10 mV for 250 ms, and then back to −67 mV for 100 ms. Dashed line indicates zero-current level. Scale Bars: 1,000 pA, 50 ms. **(D–F)** I–V relationships of ACh-sensitive currents in the three different cells as generated using the ramp protocol in the presence of 100 μM ACh. **(G–I)** Current-clamp recordings of ACh-induced changes in the zero-current potential in a torus, central zone, and planum type II hair cell during the application of 100 μM ACh (black bars).

Immunohistochemical data has shown that cholinergic vestibular efferent neurons innervate type II hair cells along the full length of the crista neuroepithelium (Jordan et al., 2015; also see Figure 1D). Previous sharp electrode recordings have shown that efferent-mediated inhibition of bouton afferents innervating medial regions (BM) is smaller and briefer than that seen in BT units, while bouton afferents innervating planum (BP) regions typically respond to efferent stimulation with a small excitation, instead of inhibition (Brichta and Goldberg, 2000b; Holt et al., 2006). We then asked whether differences in responses to ACh in type II hair cells from the central zone or near the planum could explain these variations in bouton afferent responses? We recorded from type II hair cells in all three regions (i.e., torus, central zone, and planum) and compared their ACh-sensitive currents generated with the ramp protocol as well as the effects of 100 μM ACh on the zero-current potential during current-clamp experiments (Figure 6). For this part of the study, 30 voltage-clamp and 41 current-clamp recordings were made from a total of 53 hair cells from 18 animals.

Most type II hair cells, regardless of their location showed some current inactivation with the standard step protocol (Figures 6A–C). Outward currents in type II hair cells from the planum displayed more inactivation than those from the torus and the central zone, which was consistent with previous patch-clamp recordings from dissociated turtle type II hair cells (Brichta et al., 2002). While voltage-sensitive currents in hair cells from the three regions predominantly showed fast activation and inactivation kinetics (Figures 6A,C), a few type II hair cells from the central zone (Figure 6B, *n* = 2) and torus (not shown, *n* = 16) exhibited slower activating outward components with little inactivation. An inactivation index, defined as one minus the ratio of the inactivated current at *t* = 200 ms to the peak current when the hair cell was stepped to −37 mV (Brichta et al., 2002) was calculated for each hair cell. The mean inactivation index, reflecting the percent of current lost to inactivation, was 0.29 ± 0.01 (*n* = 280) for torus type II hair cells, 0.29 ± 0.04 (*n* = 16) for type II hair cells in the central zone, and 0.41 ± 0.07 (*n* = 9) for type II hair cells near the planum. It should be noted that the mean inactivation indices for type II hair cells from three regions in this study were higher than those calculated for dissociated hair cells (Brichta et al., 2002). Small inward currents were routinely observed in all three regions.

To ensure that ACh was being delivered and establish a point of reference, we obtained ACh responses from torus type II hair cells to compare to those responses from type II hair cells in the central and/or planum regions of the same crista (Figures 6D–I). The most striking distinction was observed in our planum recordings (Figures 6F,I). In contrast to type II cells from torus ACh, failed to alter outward currents at −20 mV during the ramp protocol (cntl: 570.4 ± 221.2 vs. ACh: 578.4 ± 225.5, *n* = 6), or the zero-current membrane potential during current-clamp recordings (cntl: −50.5 ± 2.3 vs. ACh: −50.9 ± 2.3 mV, *n* = 12) in a total of 13 planum type II hair cells (sampled from a total of 4 turtles and 6 cristae), despite observing ACh responses in torus and central zone type II hair cells in the same cristae. Superfusion of a 40-mM potassium external solution was used to probe non-responders (*n* = 11) to verify that the applied solution was in fact reaching the recorded cell. That the same planum cells responded to the high-potassium external solution suggested that accessibility was likely not a contributing factor to the lack of an ACh response.

During the application of 100 μM ACh, type II hair cells from both the torus and central zone exhibited ACh-sensitive currents under voltage-clamp conditions (Figures 6D,E) and ACh-mediated hyperpolarizations in current-clamp recordings (Figures 6G,H). In the torus type II hair cell shown in Figure 6A, application of 100 μM ACh activated an outward current in voltage clamp mode which measured 479 pA at −20 mV (Figure 6D). Under zero-current, current-clamp conditions, the same cell was hyperpolarized by 34 mV when 100 μM ACh was applied (Figure 6G). In the central zone type II hair cell under the similar experimental conditions (Figures 6E,H), the corresponding ACh response values were 255.7 pA and 13.9 mV, respectively. Overall, the mean ACh-sensitive outward current measured at −20 mV was significantly larger in torus type II hair cells than hair cells from the central zone (424.7 ± 79.2 vs. 212.2 ± 35.5 pA, *p* = 0.0260, *n* = 13, 12). Similarly, the mean ACh-sensitive hyperpolarization was significantly larger in torus type II hair cells than hair cells of the central zone (−23.4 ± 2.2 mV vs. −10.8 ± 2.6 mV, *p* = 0.0002, *n* = 17, 12). With some crista preparations, the position of recorded hair cells could be identified from microscopy images taken during patch clamp recordings. Using these images, we then plotted the response amplitude to ACh from each type II hair cell as a function of linear distance along the full length of the hemicrista (~500 μm) as measured from torus to planum. Consistent with the above observations, we identified that α9^*^nAChR/SK-mediated current or voltage responses were present in only the first 250 μm (data not shown). These functional data agree with predicted longitudinal positions of turtle bouton afferents inhibited by efferent stimulation as well as *in situ* hybridization data for α9nAChR subunit mRNA (Brichta and Goldberg, 2000b; Holt et al., 2015a).

### Spontaneous presynaptic efferent activity was observed in some type II hair cells

**Figure 7:**
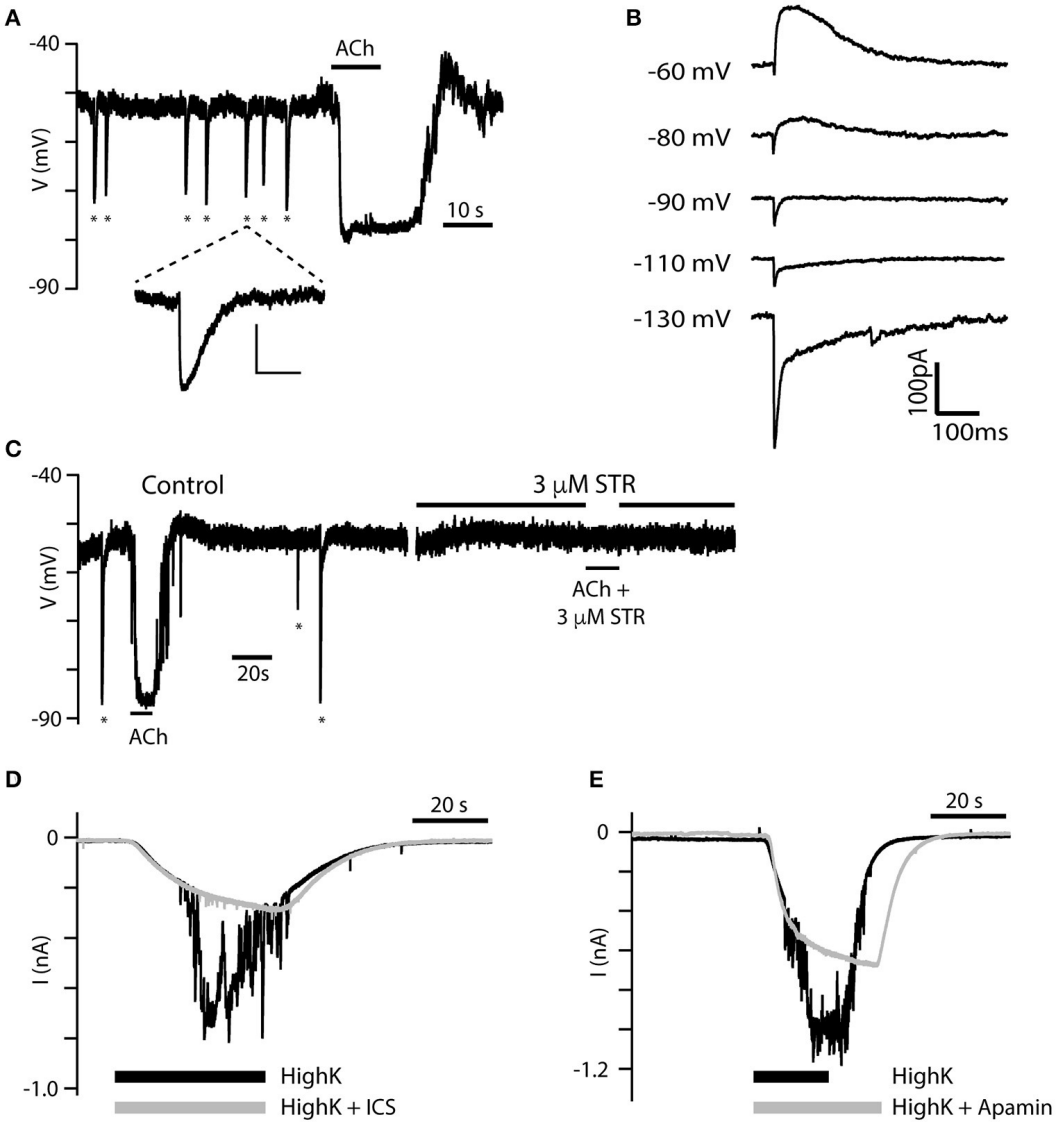
Spontaneous IPSPs reveal two components. **(A)** Spontaneous IPSPs (asterisks) were recorded during gap-free current-clamp mode from a torus type II hair cell. IPSPs were of a magnitude approaching the hyperpolarization generated by the application of 100 μM ACh (black bar). The fifth IPSP is enlarged to reveal its distinct kinetics (Scale bar: 10 mV, 500 ms). **(B)** Single IPSCs from another torus type II hair cell during voltage-clamp as the hair cell is held at different holding potentials. Two kinetic components can be identified at each potential. **(C)** Spontaneous IPSPs (asterisks) and hyperpolarizing response to 100 μM ACh were completely inhibited by 3 μM STR. **(D,E)** Currents in torus type II hair cells under gap-free, voltage clamp conditions were recorded during the application of an external solution containing 40 mM K^+^ (black) or 40 mM K^+^ external solution with either 10 μM ICS (gray, **D**) or 500 nM apamin (gray, **E**).

In 38 of 250 torus type II hair cells that responded to 100 μM ACh, spontaneous efferent synaptic activity was also observed either as large inhibitory post-synaptic potentials (IPSPs) during gap-free, zero-current, current-clamp recordings (Figure 7A), or as biphasic synaptic currents during gap-free, voltage-clamp recordings (Figure 7B). Both phenomena were observed in the absence of exogenous ACh application. Figure 7A shows a torus type II hair cell during current-clamp that displayed seven spontaneous IPSPs (marked by asterisks), followed by a large hyperpolarization triggered by the application of 100 μM ACh. These IPSPs are similar to efferent-synaptic events in other hair cell preparations, including the fast onset and slower decay. However, they fundamentally differed from the previous hyperpolarizing fluctuations seen during the washout of ACh (see Figures 2E). In fact, there was little evidence of those previously-described fluctuations in this particular cell. In voltage clamp (Figures 7B), type II hair cells with spontaneous activity were held at different holding potentials. The resulting synaptic currents were biphasic when held at −60 and −80 mV consisting first of a small and brief inward current followed by a larger and longer outward current. The initial inward current grew larger at more negative holding potentials (i.e., −130 mV) while the outward current reversed at −90 mV to give rise to prolonged inward currents. These data are consistent with the initial activation of α9^*^nAChRs followed by the subsequent recruitment of SK (Glowatzki and Fuchs, 2000; Oliver et al., 2000; Holt et al., 2006). Figure 7C shows another type II hair cell near the torus with spontaneous IPSPs and an ACh-induced hyperpolarization during control conditions, both of which were blocked following the addition of 3 μM strychnine. These observations confirm that the IPSPs, like the ACh-induced hyperpolarization, also require α9^*^nAChRs. Spontaneous IPSPs were also observed in 4 of 12 type II hair cells from the central zone that responded to ACh, but were absent from type II hair cells from the planum. Spontaneous presynaptic activities were also not observed in type II hair cells that were previously unresponsive to the application of 100 μM ACh.

In rats, the release of ACh from intact efferent terminals on hair cells can also be evoked with the application of an external solution containing higher potassium concentrations (i.e., 15–47 mM; Glowatzki and Fuchs, 2000; Oliver et al., 2000; Lioudyno et al., 2004). We also demonstrated that this approach works in the turtle split-epithelial preparation. During gap-free, voltage-clamp recordings, the application of an external solution containing 40-mM potassium induced large inward currents in torus type II hair cells held at −67 mV (*black traces*, Figures 7D,E). At their peak, these large inward currents were interrupted by a flurry of additional inward current fluctuations thought to reflect the activation of adjacent, post-synaptic α9nAChR/SK complexes, following the release of ACh from depolarized efferent terminals. Potassium concentration in the external solution is typically kept at 3 mM generating a potassium equilibrium potential of ~ −95 mV. According to the Nernst equation, elevating external potassium to 40 mM shifts the equilibrium potential to −30 mV. During the superfusion of a 40-mM potassium external solution, SK currents activated by α9^*^nAChRs would be inward. That α9^*^nAChRs and SK channels were responsible for these inward current fluctuations were confirmed by their substantial blockade following the addition of either 10 μM ICS (*gray trace*, Figure 7D) or 500 nM apamin (*gray trace*, Figure 7E). The large inward current in type II hair cells activated by the high-potassium external solution remained relatively unchanged.

## Discussion

The impetus for the current work was derived from previous studies characterizing afferent responses to efferent stimulation in sharp-electrode recordings from posterior crista afferents (Holt et al., 2006, 2015a). In those studies, cholinergic pharmacology revealed that fast efferent-mediated afferent excitation was shown to be primarily dependent on the activation of resident α4α6β2^*^nAChRs on bouton and calyx-bearing afferents while efferent-mediated afferent inhibition was attributed to the activation of α9^*^nAChRs and SK potassium channels in type II hair cells innervating bouton afferents. Although both groups of nAChRs are often recruited during efferent stimulation, it is the activation of α4α6β2^*^nAChRs and α9^*^nAChR/SK that typically dominate efferent-mediated responses in calyx/dimorphic (CD) and BT/BM afferents, respectively (Holt et al., 2006, 2015a). When monitoring afferent activity in CD and BT/BM afferents during efferent stimulation, distinguishing efferent actions on type II hair cells from efferent actions on afferent processes primarily relied on evaluating transmitter release rates and measuring response latencies (Holt et al., 2006). Consistent with a hair cell locus and the involvement of multiple synapses (i.e., efferent to hair cell to afferent), efferent-mediated inhibition of BT/BM afferents was associated with a decrease in the frequency of hair cell transmitter release and required longer than 5 ms before becoming evident in the afferent. Both phenomena were blocked by the AMPA/KA receptor antagonist CNQX providing further support that the activation of α9^*^nAChRs and SK potassium channels in type II hair cells was driving efferent-mediated inhibition. However, until now, a more direct characterization of those cholinergic efferent mechanisms in type II hair cells from the turtle posterior crista was lacking.

### Evidence for α9nAChRs in turtle type II hair cells

In the current study using patch-clamp methodologies, we recorded the current and voltage responses from over 300 type II hair cells in a semi-intact crista preparation during the application of ACh. In this preparation, ACh routinely hyperpolarized torus type II hair cells by activating a potassium conductance. This hair cell cholinergic response utilized the same synaptic mechanisms as the efferent stimulation in our sharp electrode recordings, as is suggested by similar pharmacological blockade using the α9^*^nAChR antagonists ICS, strychnine and MLA as well as the SK blocker apamin. Additional confirmation for SK channels was provided by the first reported observations of blockade with the non-peptide SK antagonist UCL1684, while the BK blocker IBTX appeared to have no effect. In our hair cell recordings, we also observed that spontaneous and potassium-evoked ACh release from intact efferent terminals shared a similar pharmacology. Our findings confirm that α9^*^nAChRs and SK channels are, in fact, expressed by type II hair cells in the turtle crista and that their activation drives efferent-mediated inhibition of BT and BM afferents. Excitatory nAChRs distinct from α9^*^nAChRs have been demonstrated in frog and bird crista hair cells, and while previous sharp electrode data have suggested that other hair cell nAChRs may be present in the crista (Holt et al., 2003, 2015a; Li and Correia, 2011), no other obvious ACh-mediated responses beyond those carried by α9^*^nAChR/SK were observed in the current study.

In our crista preparation, the dose-response curve for ACh in torus type II hair cells identified an EC50 of 76 μM, that is several-fold higher than the 10–20 μM reported for α9^*^nAChRs in dissociated hair cells from other species (Housley and Ashmore, 1991; Shigemoto and Ohmori, 1991; McNiven et al., 1996; Holt et al., 2001). It is conceivable that turtle α9^*^nAChRs are less sensitive to ACh than α9^*^nAChRs in other species, but the most likely explanation is that the higher EC50 is attributed to diffusional barriers and acetylcholinesterase (AChE) activity inherent to our semi-intact neuroepithelium, but obviously missing from isolated hair cell preparations. ACh must navigate through a number of intercellular spaces and overcome hydrolysis by AChE before arriving at sufficient concentrations to activate synaptic α9^*^nAChRs on type II hair cells. These same barriers likely contributed to the hyperpolarizing fluctuations observed in our current clamp recordings during the washout of ACh. Though beyond the scope of the current study, pretreatment with an AChE inhibitor like physostigmine would be expected to enhance the apparent sensitivity of α9^*^nAChRs in type II hair cells to exogenous ACh and lower the EC50. Such approach has worked well for the application of ACh in the frog posterior canal (Holt et al., 2003). AChE inhibitors might also shed additional light on the development and maintenance of the hyperpolarizing fluctuations.

### Sources of α9^*^nAChR-mediated hair cell depolarization

After treatment with the SK blockers apamin and scyllatoxin, efferent-mediated inhibition in BT/BM afferents was converted to excitation (Holt et al., 2006). Additional observations identified that this efferent-mediated excitation was: (1) attributed to the activation of hair cell α9^*^nAChRs no longer masked by the subsequent recruitment of SK channels, and (2) associated with substantial increase in the frequency of hair cell transmitter release that subsequently depolarized the afferent. To increase transmitter release rates, we concluded that the activation of α9^*^nAChRs either simply depolarized the hair cell to activate voltage-dependent calcium channels involved in transmitter release, or that α9^*^nAChR-mediated calcium influx and/or cisternal calcium-induced calcium release (CICR) directly engaged the synaptic machinery to promote additional transmitter release. In the current study, both apamin and UCL1684 blocked ACh-mediated hyperpolarization in current-clamp, but we failed to observe any robust ACh-mediated depolarization following blockade of SK channels. This suggested that enhancement of transmitter release, as seen in BT/BM afferent recordings, may be a product of elevations in intracellular calcium mediated by α9^*^nAChR activation and possibly CICR, as has been noted in other hair cell systems (Sridhar et al., 1997; Lelli et al., 2003; Lioudyno et al., 2004).

In vestibular and lateral line organs from bird, turtle and frog, efferent-mediated afferent inhibition is often followed by a PIE (Sugai et al., 1991; Dickman and Correia, 1993; Brichta and Goldberg, 2000b; Dawkins et al., 2005). While the convergence of inhibitory and excitatory efferent actions on different hair cells innervating single crista afferents may explain the PIE in frog (Sugai et al., 1991; Holt et al., 2003), the lack of ACh responses in turtle type II hair cells, other than those attributed to the activation of α9^*^nAChRs suggest some other mechanism must be at play. In turtle afferents, as exemplified in BM units, PIE originates in the hair cell and depends on activation of α9^*^nAChRs and SK channels, as it blocked by either α9^*^nAChR antagonists or SK blockers. These observations suggest that hair cell hyperpolarization activated a conductance that subsequently depolarized the same hair cell. In nearly 40% of our current clamp recordings from type II hair cells, the ACh-mediated hyperpolarization was followed by a pronounced depolarization which may represent the mechanism underlying PIE in BM afferents. Given the prevalence of PIE in BM afferents, it was initially surprising that the post-hyperpolarizing depolarization was also seen in current clamp recordings from torus type II hair cells. However, the extensive terminal fields of BM afferents can innervate hair cells in both the torus and central zone (Brichta and Peterson, 1994; Brichta and Goldberg, 2000a). Furthermore, our work suggests that it is likely the differences in the amplitude of ACh-mediated hyperpolarization of type II hair cells from these two regions and how they interact with the subsequent depolarization that contribute to the prominence of PIE in BM vs. BT afferents. Possible mechanisms that might account for PIE include the activation of HCN channels (Holt and Eatock, 1995; Brichta et al., 2002; Horwitz et al., 2011), the reinactivation of voltage-dependent sodium channels (Wooltorton et al., 2007; Eckrich et al., 2012), and/or the reinactivation of T-type calcium channels (Lopez et al., 1999; Martini et al., 2000; Bao et al., 2003; Nie et al., 2008) in vestibular type II hair cells. HCN channels have been previously described (Brichta et al., 2002), but convincing evidence for either sodium or T-type calcium channels in turtle crista type II hair cells is lacking. Although beyond the scope of the current study, further pharmacological tests with selective blockers should be evaluated in both sharp electrode and hair cell recordings to identify the contributing ionic mechanism(s) and to determine if PIE and the post-hyperpolarization depolarization are one in the same.

### Relating regional distribution of α9^*^nAChrs to efferent function

A second motivation for this study, that required direct recordings from hair cells, was the inference that variations in efferent-mediated inhibition of turtle bouton afferents across the crista could be attributed to differences in the functional expression of α9^*^nAChRs and SK potassium channels in the type II hair cells known to synapse on those afferents. Several studies have provided evidence for α9^*^nAChRs/SK in bird and mammalian crista hair cells (Li and Correia, 2011; Zhou et al., 2013; Yu et al., 2014; Poppi et al., 2015), but the relationship among hair cell location, α9^*^nAChRs expression and corresponding efferent-mediated afferent responses in these species has not been resolved (Dickman and Correia, 1993; Lustig et al., 1999; Li and Correia, 2011). Furthermore, using efferent-mediated response in vestibular afferents to map out α9^*^nAChRs in hair cells is particularly challenging in mammals since afferents are invariably excited by efferent stimulation (Goldberg and Fernández, 1980; Marlinski et al., 2004; Holt et al., 2015b; Mathews et al., 2017). Our data demonstrate that α9^*^nAChR-mediated changes in hair cell currents and voltages vary across the neuroepithelium, being larger in type II hair cells in the torus region than those in the central zone, while no apparent ACh responses were observed in hair cells from the planum region. While less dense than the torus region, cholinergic efferent varicosities are found in the planum abutting hair cells and BP afferents (Figure 1D; see also Jordan et al., 2015). That planum type II hair cells did not respond to ACh, at least under our experimental conditions, suggests that there may be a role for other efferent transmitters such as CGRP or ATP (Guth et al., 1998; Holt et al., 2011; Jordan et al., 2015). Since no ACh responses were noted in type II hair cells from the planum, efferent-mediated excitation of BP afferents may simply rely on the activation of α4α6β2^*^nAChRs on afferent processes in a manner similar to efferent-mediated excitation of CD afferents (Holt et al., 2006, 2015a).

Finally, recent data have suggested that the activation of the EVS, through α9^*^nAChRs, modifies both the sensitivity and discharge regularity of vestibular afferents by controlling the synaptic input of type II hair cells (Han et al., 2007; Sadeghi et al., 2007; Hübner et al., 2015; Morley et al., 2017). Changes in α9^*^nAChR activity have also been associated with shifts in the distribution of discharge regularity within sampling populations of vestibular afferents. This may have functional consequences for the integration of regularly- and irregularly-discharging vestibular afferent input necessary for driving vestibular behaviors (Hübner et al., 2015; Morley et al., 2017; Tu et al., 2017). This idea may be reinforced by the current work, in that the expression of α9^*^nAChRs in type II hair cells of the turtle crista is also correlated with afferent discharge regularity where the most irregularly-discharging bouton afferents (e.g., BT) are associated with type II hair cells showing the largest α9^*^nAChR-mediated responses. Conversely, BP afferents are the most regularly-discharging and innervate type II hair cells that appear to lack α9^*^nAChRs (Brichta and Goldberg, 2000b; Holt et al., 2015a). Although mammalian hair cells also express α9^*^nAChRs (Elgoyhen et al., 1994; Kong et al., 2005; Yu et al., 2014; Poppi et al., 2015), the relationship of α9^*^nAChR expression and discharge regularity in mammalian vestibular organs is currently unresolved.

## Author contributions

All authors (XP, DC, PJ, JH) made substantial contributions to the design of the study; All authors participated in the acquisition, analysis, and interpretation of data used in this manuscript. All authors were involved with drafting and revising the manuscript and creating the figures. All authors have approved this submission and agree to be accountable for all aspects of the work as regards its accuracy and integrity.

### Conflict of interest statement

The authors declare that the research was conducted in the absence of any commercial or financial relationships that could be construed as a potential conflict of interest.
